# Mask Use Depends on the Individual, Situation, and Location—Even Without COVID-19 Transmission: An Observational Study in Shanghai

**DOI:** 10.3389/fpsyg.2021.754102

**Published:** 2021-10-22

**Authors:** Alexander S. English, Xiaoyuan Li

**Affiliations:** ^1^Department of Psychology and Behavioral Sciences, Zhejiang University, Hangzhou, China; ^2^Intercultural Institute, Shanghai International Studies University, Shanghai, China

**Keywords:** conformity, COVID-19, mask use, observational study, social norms

## Abstract

COVID-19 has drastically altered people’s mask-wearing behaviors around the world. What is unknown is how long these mask behaviors will last post-COVID-19? To investigate how individual, situational, and locational factors influence mask use in the absence of community spread of COVID-19, we conducted an observational study in public areas in the megacity of Shanghai, China. Researchers coded people’s mask use in various suburban and urban districts and outdoor and indoor locations with and without mask requirements. Firstly, even without any local transmissions in more than 40days, 62% of the sample (*N*=1,282) still wore masks in public places. The data showed that people in more urban areas wore masks more often and that people wore masks in places where it was mandated. Women also wore masks more than men, and older people complied more with mask enforcement policies. We found that more densely populated districts and areas with more inflow of non-locals also predicted more mask use. We argue that the pandemic has long-lasting effects on human behavior like mask usage and reflects individuals’ continual conformity to new social norms.

## Introduction

The COVID-19 pandemic has immensely reshaped our understanding of urban space and health. To contain community infections, city-wide lockdowns were implemented in Wuhan, New York, London, and many other cosmopolitan cities throughout 2020. To prevent the spread of the virus, citizens were instructed to remain within their homes and neighborhoods. However, even as mandatory bans were lifted and people felt comfortable leaving their homes, people’s approach to public space and contact had been redefined by safe behaviors, and their interaction with the outside world remained the same as it had been mid-lockdown. Notably, social distancing and mask wearing have emerged as new behavioral norms (Bavel et al., 2020).

Most of the COVID-19 research has focused on the containment of the pandemic and the psychological experiences of people during lockdown; however, these studies are needed, few research studies have examined ground-level behaviors in response to health risks in public settings (Kitayama, 2020; Seitz et al., 2020). Specifically, little research has investigated non-intervention approaches (i.e., observation field studies) to truly discern the naturalistic behavior of people in their social environment in response to the pandemic (Karaivanov et al., 2021).

Our current report captures the up-to-date observations in the COVID-19 urban space by presenting data from China, the ground-zero of the global pandemic and a place where local COVID-19 community transmission was contained within 13days from the first report on January 20th, 2021. We were interested in exploring how long-lasting COVID-19 mask norms might be changing people’s social behaviors in public in response to this community outbreak. The study wanted to understand if people’s mask use might differ across individual variables (age and gender), situational factors (time of day and indoors where masks were enforced), and location (urban versus suburban settings). In the following report, we will highlight our site selection and our theoretical framework for why we explored urban mask use in post-COVID-19 Shanghai.

## Study Overview

### Why Shanghai?

Shanghai is approximately 840 KM from Wuhan and has a population of 20 million people. There are roughly 3,830 people per KM^2^ and like other global metropolises; the majority of Shanghai’s population consists of a massive inflow of internal (domestic) and international migration (Wen et al., 2010). Shanghai first reported its first COVID-19 case on January 20th, 2020, but since then, apart from the occasional local outbreak of a handful of cases in May, June, August, and November, 2020, Shanghai has been mostly COVID-19 free. As of the time of this study, Shanghai totaled only 1,828 confirmed infections with a large proportion being imported and contained from international travel. While being mostly COVID-19 free, citizens in Shanghai still might foster fairly tight mask norms. Our study provides concrete evidence of how social norms regarding masks explain and reflect different situations in which people endorse masks or certain situations in which people do not. Exploring mask norm variations within Shanghai is valuable because an intra-city look reduces confounding variables that might be present in nationwide comparisons. Looking at district-level factors and situational factors within one city also reduces potential “messier” observational data that might occur if comparing cross-cultural mask norms.

This study focuses on how individuals comply with mask use in various locations and how we might find district-level differences around the city 1year after the COVID-19 outbreak that devastated China. We especially focused on identifying the factors that may determine when, where, and why citizens wear masks or not. Observing people in the same city guaranteed a degree of homogeneity and reduced potential variation due to regional differences, city policies, and unpredictable outbreaks or changes.

### Population Density

Social contact in populated areas fosters greater chances of contracting infectious diseases (Yang et al., 2017). Recent research on mask-wearing behavior during COVID-19 has found population density as a positive correlate of virus transmission (Rader et al., 2020) and that faces masks help control transmissions (Clapham and Cook, 2021) with experts calling for mask wearing in population-dense areas (Zhai, 2020). Therefore, we assumed that areas with greater population densities may be more at risk, and people within those regions are more likely to wear masks (Haischer et al., 2020). Thus, we expected that in districts with greater population density, people would wear masks more than other areas in the suburbs.

### Mask Enforcement

After the coronavirus began to spread outside of Wuhan, China, authorities quickly adopted lockdown procedures and mask mandates to control the spread of COVID-19. Enforcement across the country was strict. In some cases, people were denied access to public facilities, detained, or punished for not following health guidelines (Beijing Daily, 2020; China News, 2020). By February 8th, 2020, Shanghai officials required citizens to wear masks outside their homes (Xinhua News Agency, 2020a). One week later, all taxi and bus drivers were required to wear masks, and people were refused service on any public transportation if they were not wearing masks (Xinhua News Agency, 2020b). Even as Shanghai became a low-risk zone and life returned to normal by the summer of 2020, masks and green health codes were still widely used and enforced in shopping malls and on public transportation (Institute for Transportation and Development, 2020). People had become accustomed to when it was time to “mask up” and when it was not necessary. Since the inception of the policy, many people complied with the requirement, while others did not. We would expect enforcement to vary across the country, but testing it within a megacity like Shanghai allowed us to eliminate the potential confusion and obscurity of inter-city mask policy differences.

### Conformity and Mask Norms

Even though mask mandates were implemented very strictly when infection risks were high, when domestic transmissions lessened and summer temperatures rose, municipal authorities loosened mask wearing (e.g., recommended indoors but not required) under certain low-risk locations Shanghai Urban (Bureau and L. E., 2020). The changing nature of mask enforcement indicates that people’s actions might not be entirely guided by policy references, and when mandates are perceptually ambiguous, people may use social cues as a reference (Burgess and Horii, 2012). Subtle social influences from the environment may serve as a guide for people to comply out of the need for self-categorization (Cialdini and Goldstein, 2004). In the classic Asch conformity experiment, researchers demonstrated the power of *groupthink*—i.e., that individuals would rather sacrifice free agency and go along with the majority to achieve group harmony. One advantage of observing people in homogeneous locations is that it allows us to explore how people in one area might respond in-sync under similar conditions, like during the unexpected spread of an infectious disease. For example, during the SARS pandemic, British researchers who attended a medical conference in Thailand observed the power of conformity and cultural assimilation of masks as they adapted to mask norms in the face of the disease threat (Syed et al., 2003). With our knowledge of COVID-19 and the power of mask wearing, we expected that most people would wear masks (i.e., a descriptive norm), and authorities would encourage or even require mask use (i.e., injunctive norm). Therefore, we contend that people would comply and wear masks more frequently where it was required and still wear masks even if not required.

### Individual and Cultural Factors

Prior to COVID-19, researchers had also studied mask use during infectious disease outbreaks, and they found disparities between gender and age groups. During SARS, more elderly people wore masks (65%) than young people (53%), and more females wore masks (66%) than men (52%; Sim et al., 2014). In a nationwide telephone study of more than 10,000 people in China, 75% (range of 64–84% across provinces) self-reported they would wear a mask if they had influenza-like symptoms (Ren et al., 2020). Shanghai reported one of the highest levels of mask adherence, at 80%. Also, a high percentage of wealthy (more than 77%) and college-educated (80%) people said they would wear masks if they were ill. While this recent study provides a valuable perspective on individuals’ mask intentions, it *only* reported people’s “intentions” to wear masks and follow other health prevention measures, not their actual behaviors in public situations.

An alternative to self-report data is to conduct a naturalistic observational study, but this approach has rarely been applied in pandemic situations. To date, one mask study during H1N1 conducted face mask observations, but it was limited to two subway stations in Mexico (Condon and Sinha, 2010). Although a similar mask study was carried out in Wisconsin, United States (Haischer et al., 2020), it was conducted in a situation where infection risks were extremely high (US total at the time of study: 7 million confirmed cases and over 200,000 deaths nationwide). It was also carried out where mask policies were not clear, and in some instances, people refused to adhere to health guidelines for political or personal reasons (Cui et al., 2021). Another cross-national study found collectivistic cultures were more likely to wear masks than individualistic cultures (Lu et al., 2021). Therefore, cross-cultural comparisons may add potential confounds due to gaps in cultural cohesion, COVID-19 knowledge, and political ideology, such as in the United States. Thus, we chose Shanghai, where social norms are relatively tight (Chua et al., 2019; Talhelm and English, 2020) as a single location of observation. To date, no such research has been carried out in China to examine the persistence of long-term mask wearing as a collective behavioral change measure despite the low risk of infection.

Therefore, the present study expands on these past studies to explore intra-city variation, situational factors (time of day and mask enforcement) which beyond basic demographic information, such as gender or age. It also allows us to gauge the long-term impact of the pandemic on social behaviors.

### Hypotheses

#### Demographic and Individual Factors (Hypotheses 1)

Even though Shanghai is a low-risk area, we hypothesized that mask usage would remain high at around 60% (H1a). This mask percentage is comparable to YouGov data from Shanghai where mask use remained over 80% from the early days of COVID-19 in 2021 (see Supplementary materials). This estimation also reflected our certainty as we observed change over time in our previous observational study at the start of the outbreak where mask use was near universal (93%) at the start of the pandemic (English et al., under review).

We expected demographic and individual variations in gender (H1b; females to wear masks more than males) and age (H1c; older people to wear masks more), both of which have been essential factors of COVID-19 risks (Haischer et al., 2020).

#### Situational Factors (Hypotheses 2)

We hypothesized that areas with mask enforcement would lead to more people wearing masks (H2a). Our point was not to prove that when mask mandates are in place, people do not have much of a choice but that the probability of deviant behavior (norm tightness) would be much lower in situations that require mask wearing than those that do not. Deviance and adherence are key to understanding conformity and norm tightness across different situations and conditions (Gelfand, 2012). In a cross-sectional study across five cities in three countries, researchers found mask use was lower with age (MacIntyre et al., 2021); however, we argue that this result depends on situations (indoor mask compliance), thus, we hypothesized that older people will comply more in mask enforced areas (H2b).

We were also interested in the idea that mask use might vary by time of day. A recent qualitative study from Tokyo, Japan speculated that nighttime mask use might decrease due to individuals acceptance of risk and more willingness to engage in deviant behaviors (Giammaria, 2020). Thus, we hypothesized that across the entire sample, nighttime mask use will be significantly lower than in daytime (H2c).

#### Locational Factor (Hypotheses 3)

We also examined the effect of city location. We expected mask usage to be generally high downtown because people commute and tour the city daily (H3). Population density is much greater in downtown areas compared to suburbs. While this hypothesis seems intuitive, a recent observation study in Hawaii found participants in downtown business districts wore masks (88% compared to 66%) significantly more than a recreational and tourism-based region (Tamamoto et al., 2020).

Finally, we explored several further predictions using district-level data that might explain mask usage. One idea is that frequent inflow and outflow of outgroup population (i.e., non-Shanghai residents) in downtown might induce locals to reduce contact by conforming to mask-wearing norms to avoid pathogen transmission (van Leeuwen and Petersen, 2018). Finally, district COVID-19 cases, population over 60, and district wealth will be incorporated into our regression models as robustness tests to verify our hypotheses that individual, situational, and locational factors can predict mask use.

## Materials and Methods

### Methodological Approach

In this study, we explore how mask differences might exist in various situations in daily life in Shanghai, China. We conducted a natural observation because it addresses the fundamental problems of self-report measures in social psychology. First, researchers have documented issues with using self-report scales due to the effect of social desirability or response style bias (Hamamura et al., 2008). This problem is persistent in the US and is just as common in collectivistic countries like China and Korea (Oyserman et al., 2002). Second, there is often a lack of correlation between personality traits (such as extraversion) and objective behaviors that tap into that same trait (Heine et al., 2008). Fortunately, observational studies can help us understand people’s real world behaviors and their psychology (Levine et al., 2001; Talhelm et al., 2018, 2019). Finally, the pandemic provides researchers a time to explore social coordination and compliance as situations to naturally they take place in the real world. While observational studies are not so procedurally as robust compared double-blind controlled experiments in the laboratory, we argue that they are a good supplement as we can observe and address real-life behaviors across social situations.

### COVID-19 Situation in Shanghai

Shanghai is the largest metropolitan city in China and is considered a model city in taming COVID-19 outbreaks even during the early, uncertain times of the pandemic (Caixin, 2020; Xu et al., 2020). It has been exceptionally effective in containing eruptions without full-scale city lockdowns or mass testing (Dezan Shira and Associates Staff in China, 2021). As of late February 2021, only 1,828 COVID-19 infection cases have been documented, with 371 being local transmissions and 1,457 “imported” from people returning from overseas (Baidu COVID-19 Data, 2021).

Over a three-day window (February 26th –February 29th, 2020), we collected 1,282 observations of mask use behavior in various parts of Shanghai. This time window and homogeneous area allowed us to study location effects, time-of-day effects, and policy effects in detail. The time of the study was more than 1month (~ 40days) since the last community outbreak in Shanghai, when a small cluster of 16 cases appeared in two districts (Huangpu and Baoshan) between January 20th and 26th (Dezan Shira and Associates Staff in China, 2021).

### Observation Rules

The researchers coded whether people wore masks, along with people’s gender and approximated age by decade (“10 and under” to “70 and above”). The researchers recorded their codes in their phone that allowed us keep digital records (see OSF file). We took precautions to ensure safety, such as wearing masks and maintaining social distance. Given the low risk of infection in Shanghai, we were also confident that the researchers were not exposed to any danger. All procedures and study design information were approved by the recommendations of the Institutional Review Board of Shanghai International Studies University IRB protocol # 2020-UNI-0211 Entitled “Psychological Experiences during the COVID-19 Outbreak.”

### Observational Settings

**Figure 1:**
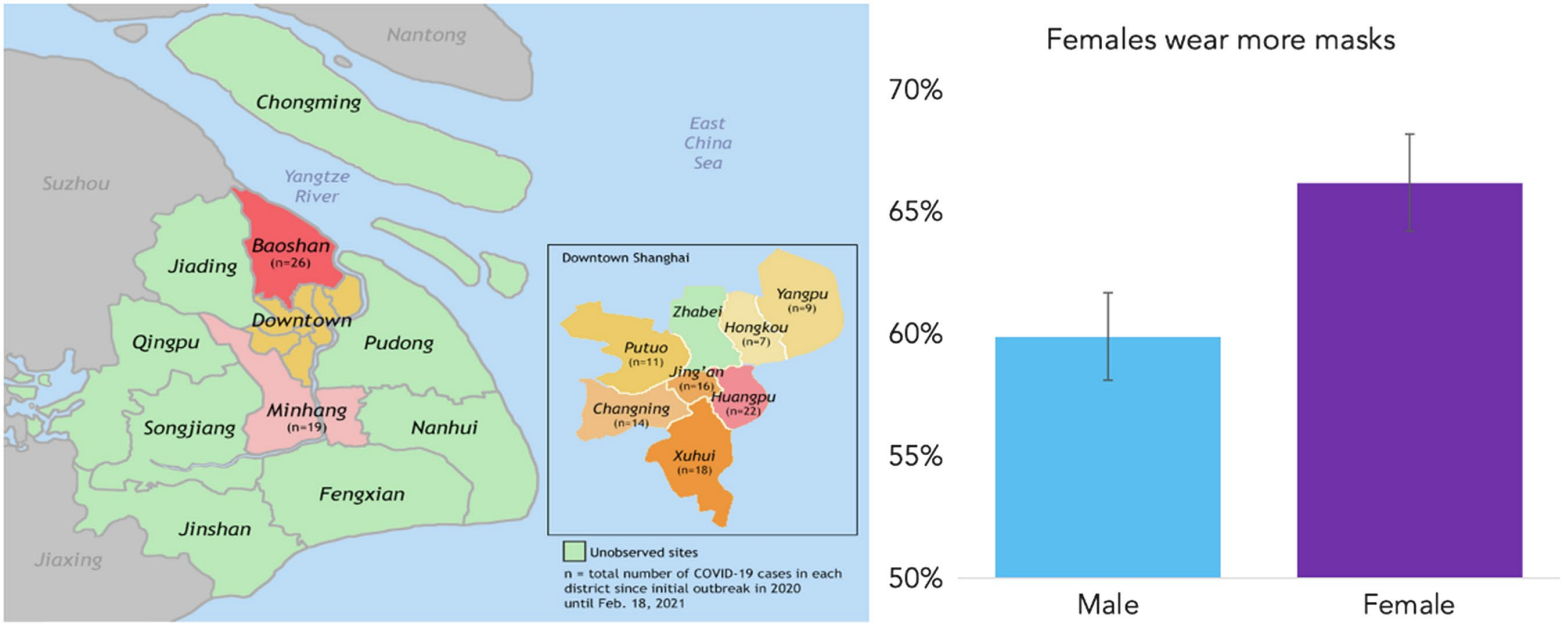
Map of Shanghai’s 16 districts with lables of total COVID-19 (*n*) cases in each district we conducted the mask observation **(left)**. Overall, 59.8% males wore masks while 66.2% female wore masks **(right)**. Error bars represent ISD.

The researchers coded people in public places, such as banks, cafés, shopping malls, outdoor community centers, and outside subway station entrances. In the analysis, we explored whether mask use differed by settings of enforced vs. non-enforced mask areas (explained in Supplementary Material). Researchers avoided tourist areas so that the data did not reflect visitors. We sampled a diverse group of people in nine districts in Shanghai (out of 16 total districts), encompassing the urban districts of Huangpu, Hongkou, Yangpu, Jing’an, Putuo, Changning, Xuhui suburban districts Minhang and Baoshan (See Figure 1 for a district map of Shanghai). These districts vary greatly in district population, density, and local COVID-19 cases. A total of 548 people (45.6%) in our sample were in the suburbs, while 54.4% (*n*=698) were located downtown (Supplementary Figure S1).

Next, we coded areas where masks were required. In total, we observed people in nine places where masks were enforced (395 observations). Because of this community outbreak, masks must be worn inside supermarkets, so the space in front of the supermarket is an area where masks are enforced. These places included supermarkets, banks, convenience stores, large indoor shopping malls, and cafés, such as Starbucks. The other areas where masks were not required were on street corners and outdoor apartment complexes. We coded mask enforcement (1=yes; 0=no), which included a body-temperature check, mask wearing upon entrance, or in some cases requiring a green QR health code to enter (described in detail in Supplementary Material).

As mentioned above, we collected our data over a three-day window to reduce the chances of a time effect or a possible eruption of new COVID-19 cases that would impact mask usage. There were no remarkable differences across the 3days. In sum, it is safe to say that our data are reliable against the possible influence of changes due to the severity of COVID-19; thus, it is a genuine representation of stability and people’s willingness or unwillingness to adhere to mask mandates even without the presence of any COVID-19 risks.

## Results

### Personal and Situational Factors

Overall, 62.71% of the people (*N*=1,282) observed wore masks. In our previous study at the start of the outbreak in January 2020, we found that older people (over 50) were less likely to wear masks (English et al., under review). This time, however, we found no difference in age groups (*p*=0.355). However, mask use was still more common among women (*B*=0.25, *z*= 2.06, *d*=0.12, *p*=0.039; Figure 1). In sum, H1a and H1c were supported, and H1b was rejected as we found no difference between age groups.

**Figure 2:**
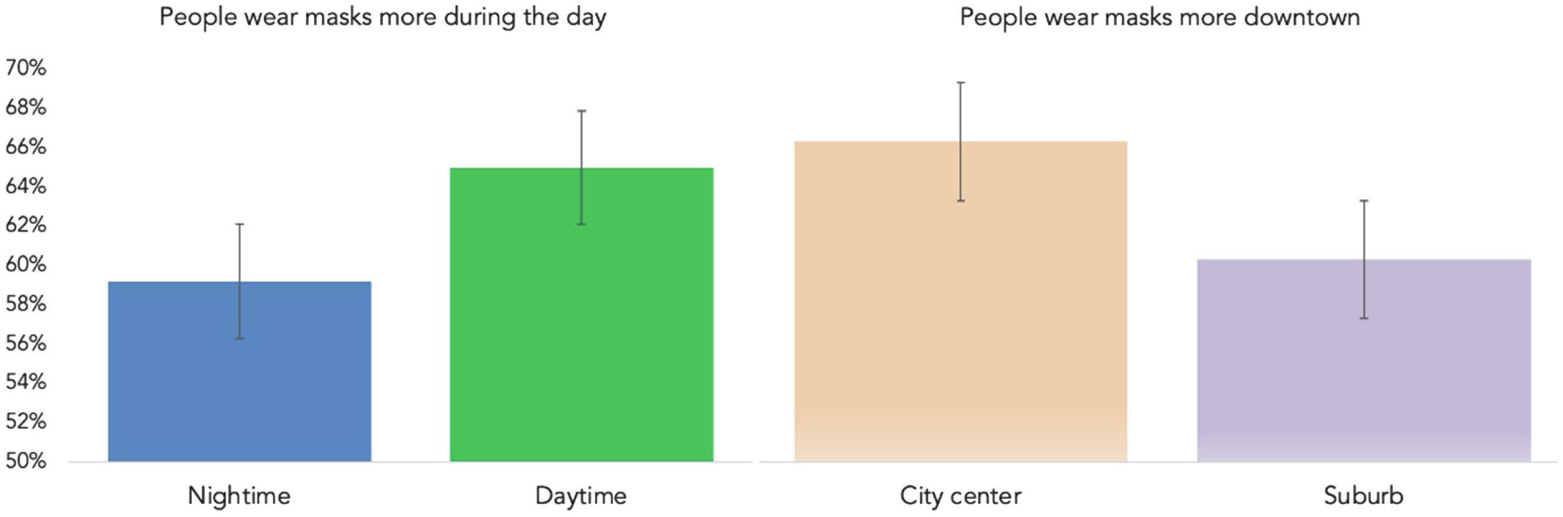
People wore masks 69.2% when in areas where mask were required as opposed to 59.6% in areas where masks were not required **(left)**. Older people conformed *more* when mask was enforced, younger people wore the same amount of masks regardless of mask regulations **(right)**.

**Figure 3:**
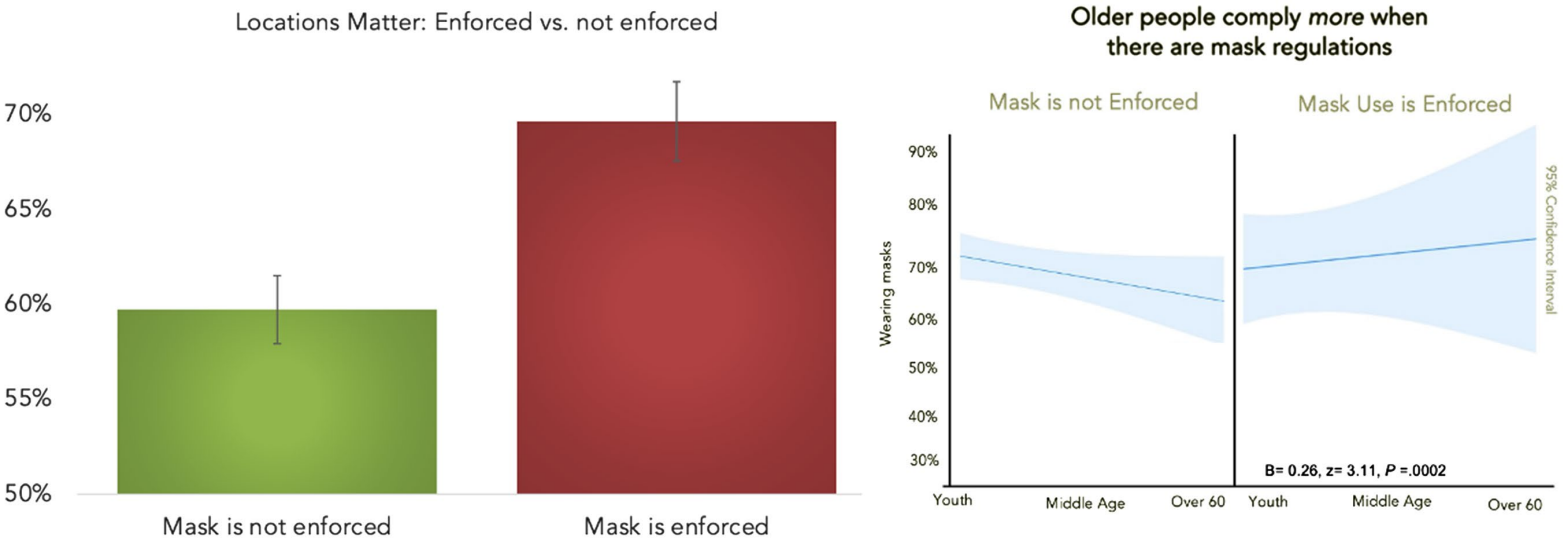
64.9% wore masks during the day compared to 59.2% at night **(left)**. 65.3% of people wore masks in the city center compared to 59.6 of in suburbs **(right)**.

As expected, across all the observation sites, mask enforcement positively predicted more mask usage (*B*=0.34, *z*= 2.43, *d*=0.14, *p*=0.015; *r*_district-level_=0.43; Figure 2). Next, since we did not find a main effect of age, it is possible that certain age groups might comply with mask mandates more. We found that age interacted with mask enforcement and that older people complied more when masks were required (*B*=0.26, *z*= 3.08, *d*=0.14, *p*=0.002; *r*_district-level_=0.43; γ represents district-level regression coefficients). This result supports previous evidence that older people comply more than middle-aged or young people (Haischer et al., 2020), but only on the condition of mask mandates (Figure 2). Finally, because we observed 792 people (62%) during the day and 490 people (38%) at night, we were able to explore the idea that people were more likely to wear masks during the day than at night (*B*=0.28, *z*= 2.18, *d*=0.12, *p*=0.029; *r*_district-level_=0.44; Figure 3). Table 1 presents linear regression models for individual and situational factors predicting mask use. In sum, H2a, H2b, and exploratory H2c were supported.

**Table 1:** Individual and situational factors that impact mask usage in Shanghai.

	Model 1	Model 2	Model 3	Model 4	Model 5
(Intercept)	0.53	0.55^+^	0.76^*^	0.30	0.30
	(0.34)	(0.33)	(0.34)	(0.22)	(0.23)
Age	−0.03	−0.03	−0.10^*^	−0.01	−0.02
	(0.04)	(0.04)	(0.04)	(0.04)	(0.04)
Female	0.25^*^	0.24^*^	0.25^*^	0.26^*^	0.25^*^
	(0.12)	(0.12)	(0.12)	(0.12)	(0.12)
Daytime (1=yes; 0=no)	0.28^*^				
	(0.13)				
Mask Enforcement		0.34^*^	−0.46		0.06
		(0.14)	(0.29)		(0.17)
Mask Enforcement x Age			0.26^**^		
			(0.08)		
Suburbs (1=yes; 0=no)				−0.24^*^	−0.44^**^
				(0.12)	(0.14)
Mask Enforcement x Suburbs					0.82^**^
					(0.27)
AIC	1662.50	1661.28	1653.63	1689.55	1673.50
BIC	1688.28	1687.06	1684.56	1710.17	1704.43
Log Likelihood	−826.25	−825.64	−820.82	−840.77	−830.75
District effects/Deviance	0.44	0.44	0.43	1681.55	1661.50

We do not nest models in the district group level because we are testing how these districts (suburbs) differ in regard to enforcement.

** *p*<0.01;

* *p*<0.05;

+ *p*<0.10.

### The Significance of Situation and Location (Expansion of Analyses)

In this section, we expand our analyses to explore more district-level factors that might be impacting mask use. For example, population across a large metropolis like Shanghai can range from densely populated high rises where millions of people live within a few square miles to huge land sprawls with only thousands of people. Thus, it makes sense that these location and situational factors might influence mask use.

We collected data from various districts to test how people in different locations might vary in their mask-wearing behaviors (Table 2). We calculated downtown districts vs. suburban districts using the city’s classification (Baidu, 2018). This gave us 584 observations in the suburbs and 698 observations in downtown areas. As expected, people in downtown areas generally wore masks more than people in the suburbs (*B*=−0.24, *z*=−2.06, *d*=−0.12, *p*=0.040). Interestingly, similar with older people, people living in the suburbs were more likely to adhere to mask enforcement (*B*=0.81, *z*= 3.08, *d*=0.17, *p*=0.002). When people were downtown and in areas with mask regulations, 67% of people wore masks compared to 74% in suburban areas where masks are enforced (Supplementary Figure S2). In contrast, people in downtown areas where masks were not enforced still wore masks 65% of the time, a sharp contrast to the 54% of people in the suburbs who were not in areas where masks were enforced.

**Table 2:** District-level factors predict mask usage.

	Model 1	Model 2	Model 3	Model 4	Model 5	Model 6
(Intercept)	2.52^***^	−0.13	0.21	0.68^*^	0.21	0.68^*^
	(0.67)	(0.24)	(0.25)	(0.28)	(0.25)	(0.28)
Age	−0.02	−0.02	−0.02	−0.01	−0.02	−0.01
	(0.04)	(0.04)	(0.04)	(0.04)	(0.04)	(0.04)
Gender	0.24^*^	0.24^*^	0.25^*^	0.25^*^	0.25^*^	0.25^*^
	(0.12)	(0.12)	(0.12)	(0.12)	(0.12)	(0.12)
Mask Enforcement	0.37^**^	0.43^***^	0.45^***^	−0.76^*^	0.45^***^	−0.76^*^
	(0.13)	(0.13)	(0.13)	(0.30)	(0.13)	(0.30)
District Population % over 60	−0.74^***^					
	(0.19)					
District Population Density		0.14^*^				
		(0.07)				
%﻿ of in-migrants _2017_			−0.002			
			(0.002)			
%﻿ of in-migrants _2017_				−0.01^***^		
				(0.00)		
Mask Enforcement x in-migrants _2017_				0.02^***^		
				(0.00)		
Urbanity					−0.003	−0.02^***^
					(0.003)	(0.00)
Mask Enforcement x Urbanity						0.03^***^
						(0.01)
AIC	1668.18	1680.47	1683.46	1665.43	1683.46	1665.44
BIC	1693.96	1706.25	1709.24	1696.36	1709.24	1696.37
Log Likelihood	−829.09	−835.24	−836.73	−826.72	−836.73	−826.72
Deviance	1658.18	1670.47	1673.46	1653.43	1673.46	1653.44

*** *p*<0.001;

** *p*<0.01;

* *p*<0.05.

We also gathered other district-level census data to verify the robustness of individual-level findings. People in districts with *fewer* people over 60years old wore masks more (*B*=−0.74, *z*=−3.86, *d*=−0.22, *p*=0.002; Table 1). This finding seems counterintuitive but suggests that districts with younger people tend to adhere to mask usage. The result resembled our previous study which found that few older adults wore masks at the start of the outbreak (English et al., under review). This might be due to the fact that areas with fewer retired people might reflect the more modernized parts of Shanghai—newer, wealthier, and busier (Zhu et al., 2019). Therefore, young white-collar workers might frequently encounter situations where masks are mandated, such as in office buildings and public transit. Second, districts with more seniors represent secure and stable populations where in-group contact is more prevalent. People might feel more security and trust toward each other in these traditional districts and lower their alertness (Wen et al., 2010).

**Figure 4:**
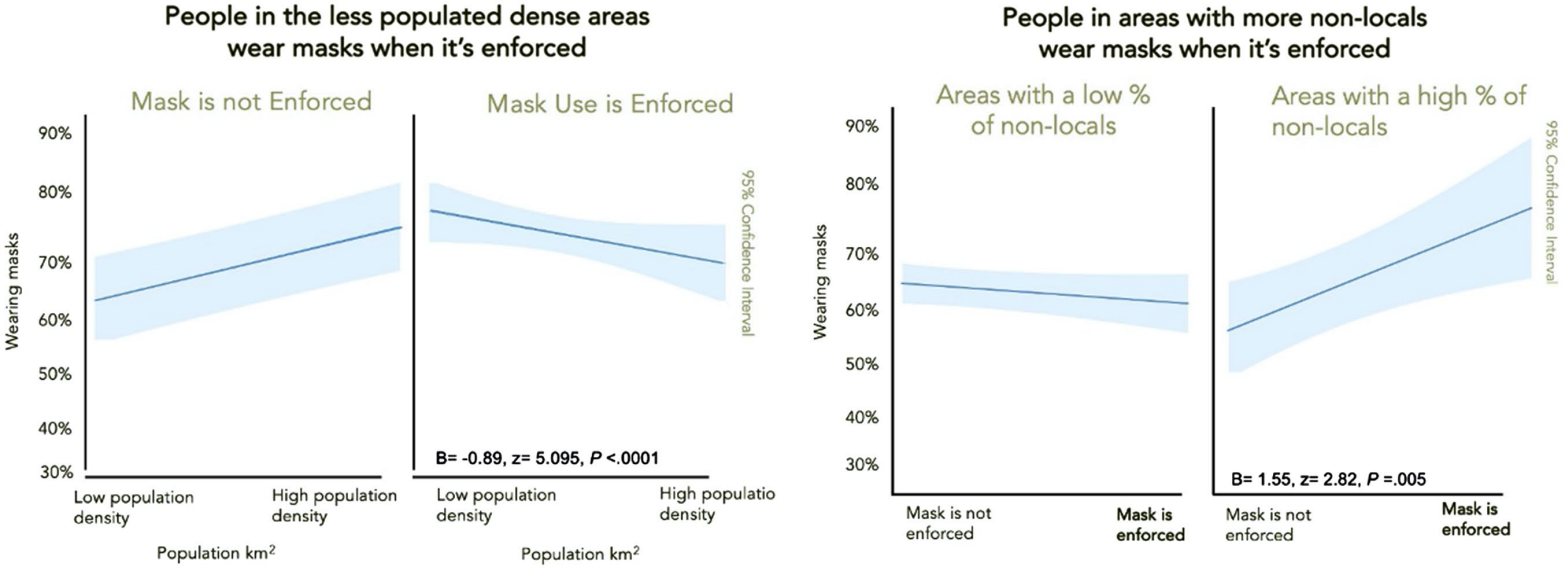
Mask enforcement mattered *less* in populated dense areas and people complied more in areas with less population density **(left)**. People wear masks more in areas with more outgroup members **(right)**.

Next, we calculated district population density based on 2019 district population census data and district land area. Population density is a logical alternative variable to the suburb vs. downtown classification. Using population density gives us more confidence (i.e., robustness test) in the results since each district has variations in population density. For example, Huangpu has a population density of 319,550 per km^2^ compared to Baoshan’s 75,360 per km^2^. As expected, people in areas that are more populated wore masks more than those in less populated areas (*B*=0.14, *z*= 2.05, *d*=0.11, *p*=0.041). Again, identical to our suburb analysis, our robustness check verified the interaction with mask enforcement; people in less population-dense areas complied more and wore masks in required areas more than people in densely populated areas where masks were enforced (*B*=−0.89, z=5.10, *d*=0.29, *p*<0.0001; Figure 4).

We also obtained statistics on the population inflow in 2017 (Shanghai Statistical Yearbook, 2018). This district-level data would allow us to tease out the total amount of non-local residents from the officially registered population in each district. While the numbers may not reflect all non-local residents, they provide a picture of which districts might represent more transient populations with people coming and going. More outgroup population inflow would mean more risk. The percent of population inflow did not predict mask usage by itself (*p*=0.26); instead, it interacted with mask enforcement (*B*=0.16, *z*=4.37, *d*=0.25, *p*<0.0001). When masks were required, people in areas with more inflow of non-locals wore masks to a greater extent than in areas with few non-locals.

Finally, following Takemura et al.' (2016) approach, we created a single district composite measure of “urbanity” by averaging population density and inflow after standardization. We found there was no main effect of urbanity on mask usage (*p*=0.266). However, it interacted with mask enforcement (*B*=0.03, z=4.37, *d*=0.25, *p*<0.0001) and followed the identical pattern as the inflow migration effect (Table 2).

Alongside population density, modernization theory would suggest that those in wealthier areas might wear masks more. We tested modern-day district GDP_2017_ (Shanghai Statistical Yearbook, 2018) and historical GDP_2007_ (Shanghai Statistical Yearbook, 2008). Neither modernization variable was significant (Table 3). Another possible factor could be COVID-19 cases. As in our previous paper, we found some evidence for base-neglect theory (Pennycook and Thompson, 2016), where individuals might pay more attention to the raw number of infected cases as opposed to the number of cases *per capita*. In a city like Shanghai, a few daily cases could be significant. Our results showed that the effect of district-level cases *per capita* was not significant (*p*=0.29), but surprisingly, total district raw cases marginally predicted *less* mask usage (*p*=0.07; Table 3).

**Table 3:** District wealth and COVID-19 cases demonstrate mixed results.

	Model 1	Model 3	Model 4	Model 5
(Intercept)	−0.20	−0.18	−0.06	0.37
	(0.29)	(0.30)	(0.25)	(0.27)
Age	−0.02	−0.01	−0.02	−0.01
	(0.04)	(0.04)	(0.04)	(0.04)
Female	0.25^*^	0.26^*^	0.25^*^	0.25^*^
	(0.12)	(0.12)	(0.12)	(0.12)
Mask Enforcement	0.38^**^	0.39^**^	0.41^**^	0.42^**^
	(0.13)	(0.13)	(0.13)	(0.13)
District GDP_2007_	0.004			
	(0.003)			
District GDP_2017_		0.002		
		(0.002)		
District Cases *_Per Capita_*			1.11	
			(1.05)	
District Total Cases				−0.02^+^
				(0.01)
AIC	1682.81	1683.43	1683.56	1681.33
BIC	1708.59	1709.20	1709.34	1707.11
Log Likelihood	−836.41	−836.71	−836.78	−835.67
Deviance	1672.81	1673.43	1673.56	1671.33

** *p*<0.01;

* *p*<0.05;

+ *p*<0.10.

## Discussion

The purpose of this observational study was to understand various conditions in which individuals would adhere to mask usage. We focused entirely on the metropolitan city of Shanghai because it was at considerable risk early during the pandemic (Zhang et al., 2020) and was later praised for its model role in taming transmissions. Using this urban example, we examined how conformity concerning mask-wearing behaviors shaped the city’s population 1year after the initial outbreak. We found that despite low infection risks, of the 1,282 people observed in Shanghai, 62.7% still wore masks regardless of location, time of day, or district. These results suggest that long-lasting COVID-19 behavior norms are likely to persist well into the future, especially in countries where vaccine rollout is slow. We went beyond other mask studies investigating responses during the early outbreak across different Chinese regions by researching district-level factors that might impact people’s compliance (English et al., under review); (Haischer et al., 2020). Integrating our findings, we argue that compliance with mask requirements reflects voluntary conformity to COVID-19 norms due to urbanization factors and COVID-19 prevention.

Our observations yielded a number of novel results that deserve discussion. First of all, our findings are consistent with previous research (Haischer et al., 2020) that females conform more to mask wearing. However, we found no noticeable age difference in mask usage. We replenished previous research by finding that mask wearing is more prevalent during the day than at night. Moreover, age interacted with mask enforcement in predicting mask wearing, suggesting that older people may wear masks because they are required to. In fact, we find that young people wear more masks even *without* enforcement. Put another way, young people’s mask-wearing behavior is less influenced by mask rules. These findings correspond with previous cross-country research using extensive data from YouGov from 27 countries which found that people over the age of 50 tended to comply with COVID-19 social distancing measures (Daoust, 2020). Interestingly, the author excluded data from China because the data were not representative. Our data suggest that this trend could be dependent on country-level specificities and is not universal.

Secondly, both situations (whether masks are enforced) and location (suburb vs. downtown) matter for mask conformity. Tests of the interaction between the suburb/downtown dichotomy and district population density with mask enforcement have provided substantial evidence to confirm that mask enforcement is more powerful in suburban areas in achieving mask compliance. Most notably, the percentage of urbanites wearing masks did not differ much whether masks were enforced (67%) or not (65%). These findings suggest that while mask conformity for suburban residents is still susceptible to external factors, such as mandates, masks may have become a behavioral norm for urban dwellers.

Thirdly, we found that districts with a younger population predict more mask usage. This finding, corresponding with the fact that urban districts in Shanghai are home to young white-collar workers (Liu et al., 2015), is consistent with the above finding that mask wearing may have become normative in urban areas.

It is also not surprising that greater population inflow interacted with mask mandates to achieve mask wearing, as inflow migration suggests more outgroup contact, for example, these places tend to have more mobility and inbound and outbound traffic, like as train stations, bus stations, or airports. For clarification, the greatest population inflow in Shanghai represents both urban and suburban areas, such as Yangpu (16,485 in 2017) and Minhang (15,098 in 2017); hence, the interaction with mask enforcement does not counter our previous finding on urbanization and mask conformity. Similarly, Shanghai’s wealth is not solely built upon urbanized locations—our major suburban site, Minhang, contributed the greatest amount of GDP in 2017. Since GDP is not a marker of urbanization in our study, it seems logical as to why wealth did not predict mask use.

Finally, only raw cases marginally predicted mask usage but in the *opposite* direction of what would be expected. Perhaps this is because most places in Shanghai have not had local transmissions since February 2020. People city-wide have adopted a behavioral mask norm regardless of district case transmissions. Or, this could be due to the fact that infected cases and their close contacts were put under instant quarantine in Shanghai, and it is possible that people contradictorily felt safer going out even without a mask given reduced threat and uncertainty from accessing public tracking information (Wnuk et al., 2020).

## Conclusion, Limitations, and Future Directions

By conducting observational research on mask wearing over a three-day window in Shanghai, China, the current study examined conformance with mask norms in the ongoing COVID-19 pandemic. An observational approach allowed us to tease out the confounding factors in a traditional survey design and enabled us to disentangle the demographic, situational, and locational impact on mask norms. While our study demonstrated the unsurprising role of mask enforcement in strengthening norms, it also revealed how the urban environment especially fosters new norms that may last into the future.

The observational design is also a limitation of the study. First, the short duration of time limited us from truly unpacking the formation of norms as a process. Second, since we were unable to observe equally (according to location and time of day) across all nine districts, it is possible that there might be some bias or confounding variable at play due to variation in time or location in various districts. Third, researchers also observed locations that were close or nearby their residence or a place they frequented often, this might provide some unknown bias and can be seen as an important limitation. Our sample city, Shanghai, is also different from many other cities in China and the world given its unique administrative and internationalized culture. Therefore, relevant findings may pose limitations to other urban settings due to macro-level factors not included in the study. Unlike other studies, we did not observe mask-wearing behaviors that are technically not protecting people from infection risks and not adhering to health guidelines. Still, our focus is on mask-wearing norms and willingness to follow mask policies, not on how effectively masks are used and whether they are worn properly or not.

## Conclusion

Our research captured what norms may look like in urbanized settings if COVID-19 becomes the “new normal” of the present era. By utilizing observations from the “model” city of Shanghai in containing this pandemic, we demonstrated that norms are the co-product of enforcement and shared collective responses to protecting others (English et al., under review). Our results prove that what the Shanghai municipal government coined as “normative administration” in its daily press release (The State Council Information Office of the PRC, 2020) has truly taken effect on the societal level. Our findings may serve valuable on the ground observations on how large cities might better tackle the current and future pandemic to increase coordination and compliance with health mandates.

## Data Availability Statement

The original contributions presented in the study are included in the article/Supplementary Material, further inquiries can be directed to the corresponding author.

## Ethics Statement

The studies involving human participants were reviewed and approved by Shanghai International Studies University Institutional Review Board (IRB) Research Project Protocol # 2020-UNI-0211 Entitled “Psychological Experiences during the COVID-19 Outbreak.” Written informed consent from the participants’ legal guardian/next of kin was not required to participate in this study in accordance with the national legislation and the institutional requirements.

## Author Contributions

AE and XL designed the study, collected and coded data, and wrote the paper. AE analyzed the data. All authors contributed to the article and approved the submitted version.

## Conflict of Interest

The authors declare that the research was conducted in the absence of any commercial or financial relationships that could be construed as a potential conflict of interest.

## Publisher’s Note

All claims expressed in this article are solely those of the authors and do not necessarily represent those of their affiliated organizations, or those of the publisher, the editors and the reviewers. Any product that may be evaluated in this article, or claim that may be made by its manufacturer, is not guaranteed or endorsed by the publisher.
