# Supplementary material for: Mask Use Depends on the Individual, Situation, and Location—Even Without COVID-19 Transmission: An Observational Study in Shanghai
Source: Front Psychol. 2021 Oct 22;12:754102. doi: 10.3389/fpsyg.2021.754102 (PMC8569386; doi:10.3389/fpsyg.2021.754102)
Supplement: Supplementary file 1 [file Data_Sheet_1.doc]

**Mask Use Depends on the Individual, Situation and Location— Even Without COVID-19 Transmission: An Observational Study in Shanghai**

**Alexander S. English**

**Xiaoyuan LI**

Supplemental Information


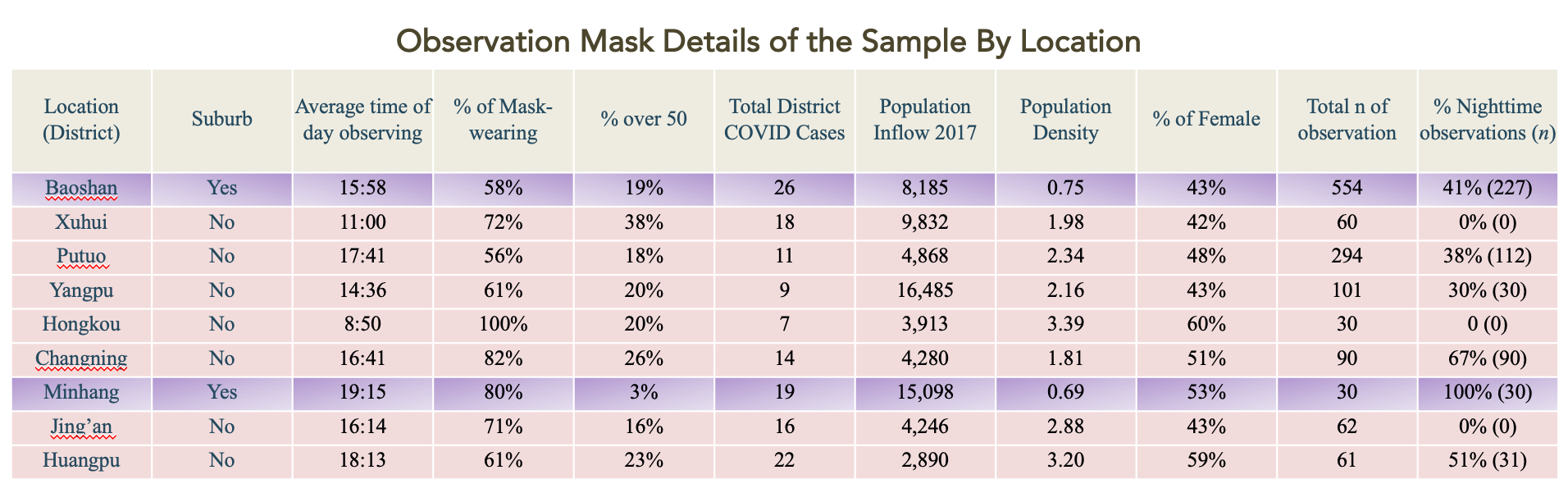


Figure S1. Basic demographic features of the observation sites across Shanghai. Purple rows refer to Suburb areas while Pink rows refers to downtown districts . Average time of day observing reserves to the average based on observations. In some locations, researchers observed multiple times. Nighttime observations were conducted after sunset in the given location. In total there were 395 nighttime observations.


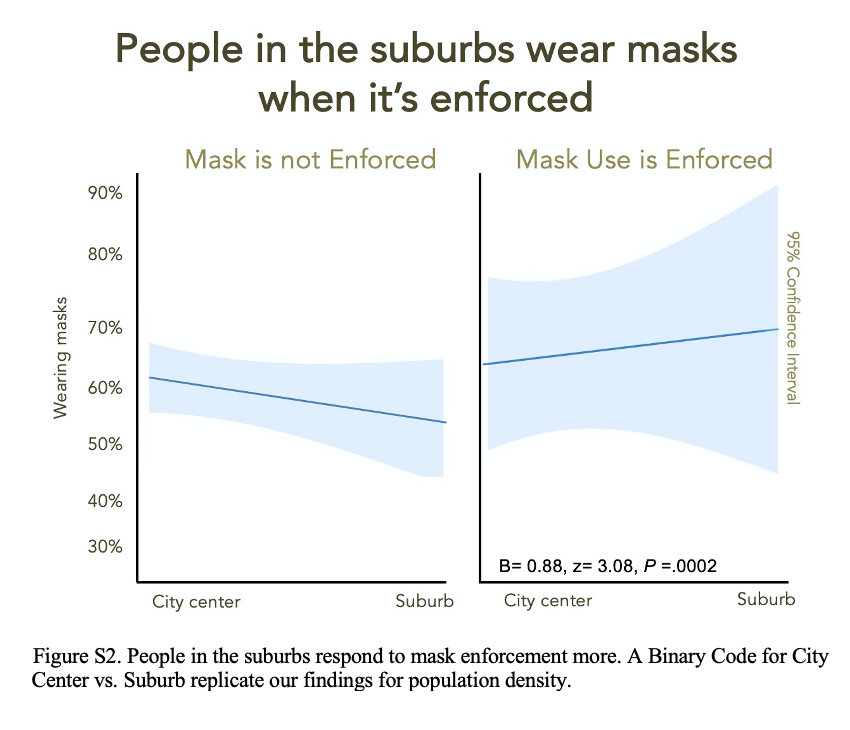


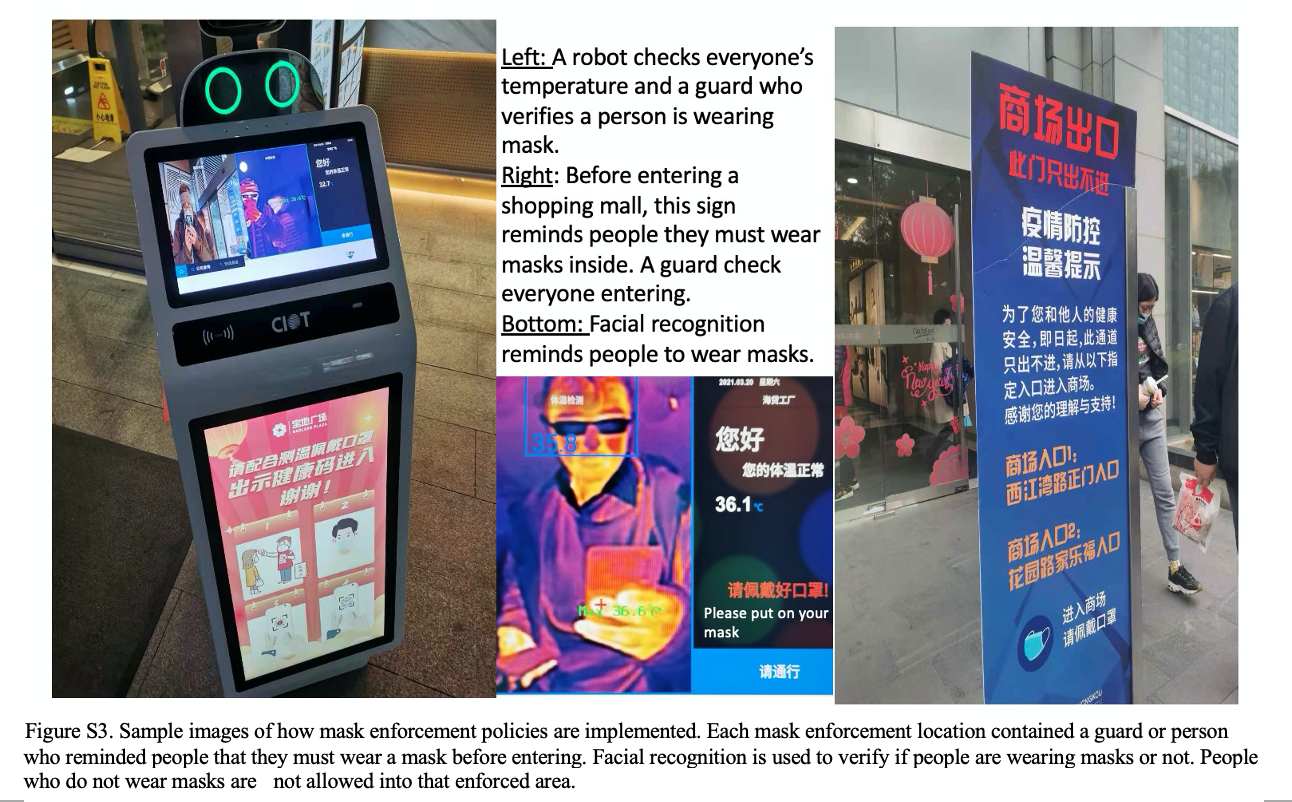


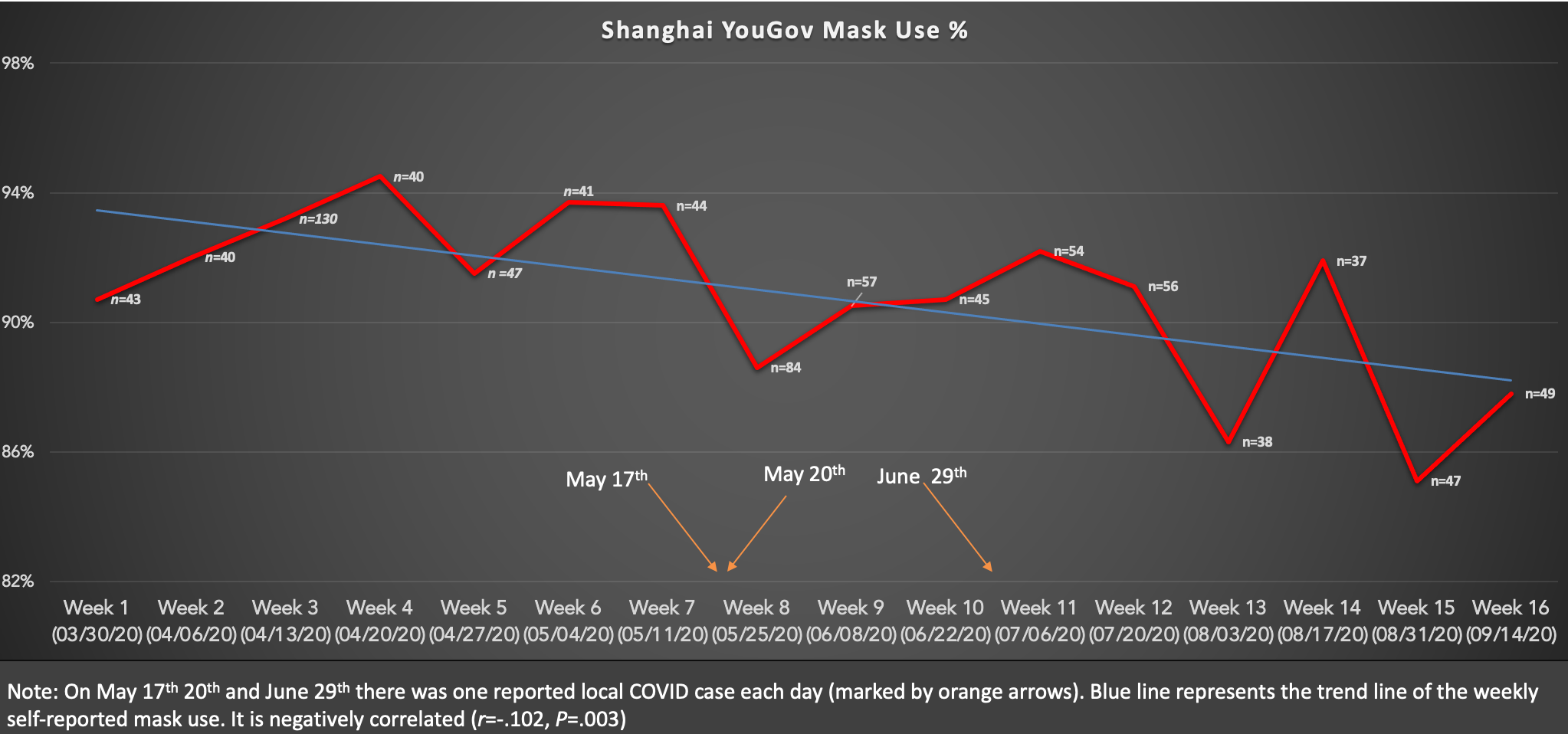


Figure S4. Authors sourced the YouGov Data to explore how people in Shanghai responded to a weekly self-reported mask use frequency question. In general, mask use declined slightly over time from over 91% to around 88 % over time. We recoded the mask use to (0=0%, 1=20%, 2=40%, 3=60%, 40=80%, 5=100%) for easy interpretation. The orange lines and data represent a time when one new local COVID infection was reported in Shanghai.

Supplemental Information

In this section, we provide additional analyses and explanations of key terms and models we used to support our claims in the main paper.

**S1. Statistical Analysis.** We tested the key findings in both regressions and hierarchical linear models, which nested observations in districts. We conducted logistic regressions (1 = wearing mask, 0 = no) using the GLMER function (generalized linear mixed effects) and GLM function in the program R. The GLMER function in R does not provide effect size estimates. To calculate effect sizes, we used the change in the district-level variance (pseudo-*R^2^*) of the model with and without the key predictor. We took the square root of this to get the correlation *r*, which is a more familiar effect size.

**S2. Power and Calculating Effect Sizes in GLM.**

At the convention for a small effect size (Cohen’s *d* = 0.2), the mask observation study had 96% statistical power. We sampled areas where COVID-19 cases varied, ranging from high (*n*=26) to low (*n*=9). The GLM function in the program R does not provide standardized effect size estimates at the individual level. To give readers an idea of effect sizes, we converted t and z values into Cohen’s *d*.

**S3. Mask Enforcement.**

Mask enforcement is common in all areas in China and most countries in the world now. In China, these places usually represent indoor spots with heavy foot-traffic and contain a lot of densely populated areas. In this observational study, all enforced areas were indoors and had someone at the door or a robot verifying mask usage when entering ([Figure S3](#FigureS3)). We coded people inside these areas once they entered and passed the enforcement location. Codes were 1 = yes and 0 = no and represented locations of mask enforcement.

**S4. Time of Day.**

Across each district, we recorded the observation time and noted the location. Our observations ranged from 8:50 am to 21:30 at night. This allowed us to explore the role of time in predicting people’s likelihood to wear masks at different time of the day. In the main text, we used a daytime and nighttime variable (binary) based on the day of sunset. On February 26^th^, sunset was at 17:50, on February 27^th^ and 28^th^ , sunset was at 17:51. This accuracy allowed us to recode observations accordingly. While we are confident with our results, it’s possible that a binary variable might not capture the diversity of mask use throughout the day. Perhaps early in the morning and late at night, people will wear masks at different rates. We reran analyses as in Table 1, with time of observation, controlling for age, gender and mask enforcement. We ran a linear regression and found time of observation was negatively correlated with mask use (*B =* -0.0005, *t* =-4.59*, P* <0.001). Unfortunately, not all districts had daytime and nighttime mask observations. We include the variation of observations according to each district (Figure S2).

**S4. YouGov Shanghai Sample.**

According to the YouGov tracker, over 80% people in China wore their masks in public places between Feb 23^rd^ and Oct 11^th^ 2020 (<https://yougov.co.uk/topics/international/articles-reports/2020/03/17/personal-measures-taken-avoid-covid-19>). While this represents the entire country, the data points to the overall majority of people surveyed who endorse mask usage, even without the presence of infections in their local communities. Our interest is in Shanghai and in order to verify some predications we made in our mask observation study (Study 1), we sourced the raw data from YouGov Imperial College (<https://github.com/YouGov-Data/covid-19-tracker>). The data was collected in Shanghai over 16 week period March 30^th^, 2020 and September 14^th^, 2020. This subsample included 852 respondents from Shanghai (*M*_age_=35.16, *SD* = 11.28) with 420 females (49.3%). This dataset only included these demographic variables and one question regarding mask frequency. Participants were asked: “How often have you worn a face mask outside your home (e.g., when on public transport, going to a supermarket, going to a main road)?” (0 = not at all, 1 = rarely, 2 = sometimes, 3 = frequently, 4 = always).

Linear regression results from the Shanghai sample showed that female respondents wore masks significantly more than men (*B*= 0.25, *t* = 4.61,*d* =0.32 , *P* <0.001) and overtime mask use significantly decreased (*B* = -0.02, *t* = -3.02, *d* = -0.21, *P* = 0.003). Finally, mask use did not vary by age (*P* = 0.190). Unfortunately, there was not question to indicate where they lived in Shanghai nor their likelihood of complying with mask requirements when going indoors. Finally, we graphed the slight decrease in mask use over in Figure S4.
